# The Signaling Duo CXCL12 and CXCR4: Chemokine Fuel for Breast Cancer Tumorigenesis

**DOI:** 10.3390/cancers12103071

**Published:** 2020-10-21

**Authors:** Karolina A. Zielińska, Vladimir L. Katanaev

**Affiliations:** 1 Translational Research Center in Oncohaematology, Department of Cell Physiology and Metabolism, Faculty of Medicine, University of Geneva, 1211 Geneva, Switzerland; Karolina.Zielinska@unige.ch; 2 School of Biomedicine, Far Eastern Federal University, 690090 Vladivostok, Russia; 3 Institute of Oceanography, Minjiang University, Fuzhou 350108, China

**Keywords:** breast cancer, chemokines, chemokine receptors, CXCL12, CXCR4, SDF1

## Abstract

**Simple Summary:**

Breast cancer remains the most common malignancy in women. In this review, we explore the role of the CXCL12/CXCR4 pathway in breast cancer. We show that the CXCL12/CXCR4 cascade is involved in nearly every aspect of breast cancer tumorigenesis including proliferation, cell motility and distant metastasis. Moreover, we summarize current knowledge about the CXCL12/CXCR4-targeted therapies. Due to the critical roles of this pathway in breast cancer and other malignancies, we believe that audiences in different fields will find this overview helpful.

**Abstract:**

The CXCL12/CXCR4 signaling pathway has emerged in the recent years as a key player in breast cancer tumorigenesis. This pathway controls many aspects of breast cancer development including cancer cell proliferation, motility and metastasis to all target organs. Moreover, the CXCL12/CXCR4 cascade affects both immune and stromal cells, creating tumor-supporting microenvironment. In this review, we examine state-of-the-art knowledge about detrimental roles of the CXCL12/CXCR4 signaling, discuss its therapeutic potential and suggest further research directions beneficial both for basic research and personalized medicine in breast cancer.

## 1. Breast Cancer: The Most Common Invasive Cancer in Women

Breast cancer remains the most common malignancy in women. Although early stage non-metastatic breast cancer is curable in approximately 70–80% of patients, advanced disease with distant metastasis currently remains incurable and accounts for approximately 90% of deaths [1,2,3]. Management is challenging due to the complex molecular biology of the disease. Breast cancer is a heterogeneous disease with involvement of hormone receptors (estrogen (ER) and progesterone (PR)), human epidermal growth factor receptor 2 (HER2, encoded by ERBB2), Wnt signaling receptors and/or BRCA mutations [2,4,5,6,7].

Initial classification from the year 2000 distinguished four breast cancer subtypes, but currently a surrogate classification with five subtypes is used in the clinic. Depending on the receptor expression and histological features, breast cancer can be divided into luminal A-like, luminal B-like HER−, luminal B-like HER+, HER2-enriched (non-luminal) and triple negative breast cancer (TNBC) that tests negative for ER, PR and HER2 [2,8]. HER2 is overexpressed in approximately 20% of breast cancers and correlates with poor prognosis in the absence of systemic therapy. TNBC constitute approximately 15% of all breast cancers and the pathological processes underlying this malignancy remain poorly understood [3].

The metastasis pattern in breast cancer depends on the subtype. Luminal A tumors tend to relapse within five years of first presentation, similar to luminal B and HER2− malignancies, and they metastasize mainly to the bone and lymph nodes, whereas HER2+ tumors metastasize to the brain. TNBC tumors usually relapse early, within 2–3 years of first presentation and have tropism for lungs and brain [2,3].

Therapeutic options depend on the disease subtype and include surgery, radiation therapy and systemic approaches such as chemotherapy, endocrine therapy and immunotherapy; recently great effort has been made to deescalate the treatment and limit adverse side effects. Estrogen and progesterone receptors are two main molecular targets in breast cancer pathogenesis [2]. Endocrine agents have been used systemically to inhibit ER signaling in ER+ and PR+ tumors [3,9]. In early HER2+ breast and TNBC, cancer neoadjuvant therapy remains a common choice [5,9]. Although TNBC, the deadliest subtype of breast cancer, currently lacks targeted therapies, novel promising drugs are on their way to the clinic [4,10]. Therapy for patients with advanced metastatic breast cancer aims to prolong survival and control the disease with low treatment associated toxicity [2].

## 2. Chemokines Are Key Regulators of Tumorigenesis

Chemokines are chemotactic cytokines and can be divided into four subtypes: XC, CC, CXC and CX3C chemokines depending on the position and number of cysteine residues in the N-terminus. Chemokine receptors belong to the G protein-coupled receptors (GPCRs) family and typically bind to more than one chemokine [11,12,13]. They are also divided into four groups corresponding to the chemokine classification: XCR, CCR, CXCR and CX3CR. Moreover, chemokines bind atypical chemokine receptors (ACKRs) that can act as scavenger receptor [14].

Chemokines control cell movement both during homeostasis and inflammation, and cells that express specific receptors migrate along chemokine gradients [11,15]. Some chemokines can also exert cytokine-like actions on cells, e.g., stimulating cell proliferation [11]. Moreover, chemokines have been implicated in a number of diseases including autoimmune diseases, viral infections and cancer [15,16,17].

In cancer, chemokines together with their receptors affect tumor development and metastasis [17,18,19,20]. Activation of chemokine signaling induces transcription of the target genes that promote motility, cell invasion, interactions with the extracellular matrix (ECM) and cell survival [11]. Since tumor infiltration by immune cells is crucial to elicit proper anti-tumor response, chemokines also play an important role in guiding immune cells into the tumor microenvironment. Specific chemokine receptors are expressed on both immune and tumor cells and their expression on cancer cells correlates with disease prognosis [21].

## 3. The CXCR4 Chemokine Receptor Controls Multiple Aspects of Tumorigenesis

CXCR4 is the most common chemokine receptor expressed on malignant cells; it has been found in more than 23 human tumor types such as kidney, lung, brain, breast and prostate cancers [11,22]. This receptor is mostly detrimental and has been implicated in cancer progression and dormancy of cancer cells [22,23]. In prostate cancer, CXCR4 overexpression is associated with an aggressive phenotype and poor overall survival. Similarly, CXCR4 signaling in leukemia enhances invasion and induces therapy resistance. Interestingly, multiple myeloma seems the only malignancy where CXCR4 overexpression improves treatment sensitivity and provides longer median survival time [24].

CXCR4 has been the first chemokine receptor found hijacked by breast cancer cells to proliferate and metastasize to distant organs [25]. In patients with breast cancer, CXCR4 overexpression is associated with the lymph node status and poor prognosis [25,26,27]. Furthermore, almost 75% of patients with TNBC show high CXCR4 expression [24,28].

Moreover, CXCR4 is expressed on many cell types including both hematopoietic cells such as leukocytes and progenitor and stem cells and non-hematopoietic cells including endothelial and stromal cells, which play important roles in tumorigenesis [15]. Several factors such as vascular endothelial growth factor (VEGF), hypoxia and estrogen can induce transient expression of CXCR4 on cells that normally do not express this receptor [11,29].

Two CXCR4 splice variants have been described: CXCR4-A and CXCR4-B. The latter is expressed more often and has been shown to form both homo and heterodimers (with other receptors such as ACKR3). This process modifies signal transduction [15].

CXCR4 has only one chemokine ligand CXCL12 (also known as stromal cell-derived factor 1 (SDF 1)), which is uncommon in the world of chemokine signaling [15]. Although CXCL12 has initially been described as a homeostatic chemokine controlling angiogenesis, hematopoiesis and embryogenesis, a growing number of studies suggests its contribution to diverse pathologies including cancer. In cancer, it mediates primary tumor growth, metastasis, chemoresistance and angiogenesis [15,30,31]. Specifically, CXCL12 seems to play a detrimental role in breast cancer; its expression in patients has been extensively studied. High CXCL12 levels are present in patients with lymph node and brain metastasis and inversely correlate with the overall survival [32,33,34]. CXCL12 in breast cancer is typically released from stromal cells, and in TNBC it is secreted by a specific immunosuppressive subset of fibroblasts [33,35].

## 4. Transcriptional Regulation of CXCR4 and CXCL12 in Breast Cancer

Several transcription factors have been reported to regulate the expression of CXCR4 and CXCL12 in breast cancer cells and those transcription factors can cooperate. For example, hepatocyte growth factor activates ERK1/2 and subsequently Ets1, which binds to DNA and in cooperation with NF-*κ*B induces CXCR4 transcription. Under hypoxia, NF-*κ*B and hypoxia-inducible factor-1 (HIF-1) remain crucial inducers of CXCR4 [36]. Moreover, NF-*κ*B alone can activate the expression of CXCR4 in metastatic breast cancer cells via direct interaction with its promoter inducing cell motility [37].

KLF8 has been implicated in the regulation of CXCR4 signaling and disease progression. KLF8 binds to and activates the CXCR4 promoter and its knockdown in MDA-MB-231 cells decreases migration, invasion and transendothelial migration towards CXCL12. Moreover, patient data analysis shows that overexpression of KLF8 and CXCR4 correlates with metastatic potential [38]. Likewise, transcription factor forkhead box C1 (FOXC1) induces the expression of CXCR4 and enhances migration of MDA-MD-231 cells and lung metastasis in vivo [39]. In addition, POU domain class 2 transcription factor 1 (known as POU1F1 (Pit-1)) induces the expression of both CXCR4 and CXCL12 in breast cancer cells [40].

The GLI transcription factors—the effectors of Hedgehog signaling—also play an important role in breast cancer progression. GLI1 and GLI2 upregulate several genes implicated in CXCL12 signaling such as CXCR4, ACKR3 and actin binding protein LCP-1/L-PLASTIN. GLI1 binds to the CXCR4 promoter and induces CXCR4 transcription. Moreover, GLI1 also stimulates CXCL12-dependent ERK phosphorylation and migration of breast cancer cells [41]. Another study showed that a C-terminal binding protein (CtBP)-dependent transcriptional repressor TSHZ2 of GLI1 is downregulated in breast cancer cell lines leading to overexpression of GLI-target genes such as CXCR4 [42].

Members of the nuclear receptor superfamily such as estrogen receptor (ER) *α* and *β* also regulate the transcription of the CXCL12/CXCR4 cascade components in breast cancer. Challenge of MCF-7 cells with CXCL12 enhances the transcriptional activity of ER and expression of its target genes including CXCL12 due to the positive feedback between CXCL12 signaling and ER-dependent gene transcription [43]. Similarly, another study found that estrogen enhances both the expression of CXCL12 and CXCR4 via distal ER binding sites located 20-250 kb from the transcription start site [44]. CXCL12 also enhances ER-mediated gene transcription via activation of ERK1/2 and p38 [45].

## 5. The CXCL12/CXCR4 Signaling

Upon CXCL12 binding to CXCR4, GPCR-mediated signal transduction begins and G*βγ* and G*α* subunits dissociate from each other [15]. G*α* subunits regulate the core properties of G protein signaling and engage specific signaling effectors. CXCR4 can couple to different isoforms of the G*α* subunit, determining the downstream response.

G*α_i/o_* controls CXCR4-mediated Rac activation, chemotaxis and adenyl cyclase inhibition [46,47]. Furthermore, G*α_i_* induces migration of non-metastatic breast cancer cell lines whereas migration of metastatic breast cancer cells remains G*α_i_* independent [48]. In contrast, another study showed that, due to low G*α_i_* expression in non-metastatic cells, CXCR4 remains active only in metastatic breast cancer cell lines [49].

Although CXCL12-induced migration in CXCR4-expressing cells is typically pertussis toxin-sensitive and thus involves G*α_i/o_* proteins, other G*α* proteins might participate in the chemotaxis. For example, migration towards CXCR4 ligands in DCs also depends on G*α_q_* [50]. Moreover, CXCR4 can couple to G*α*_13_ and activate a wide range of effectors such as Rho and induce cell migration upon challenge with CXCL12 [46,47]. Similarly, the CXCR4-mediated chemotaxis and trans-endothelial migration of metastatic breast cancer cells depend on G*α*_13_ and Rho [48].

Engagement of CXCR4 and G proteins leads to the activation of several downstream signaling cascades such as MAPK, phosphatidylinositol 3-kinase and calcium release mediated by phospholipase C and NF-*κ*B [15,51]. For example, CXCR4 activation induces FAK, ERK and Akt signaling in pancreatic cancer cells and boosts transcriptional activities of *β*-catenin and NF-*κ*B. Moreover, it induces the expression of survival proteins [52]. In ovarian cancer cells, CXCR4 induces MAPK activation and cell proliferation [53].

*β*-arrestin can also be recruited to CXCR4 upon its activation by CXCL12 enhancing signal transduction or inducing receptor internalization; most GPCRs use both *β*-arrestin and G protein signaling pathways [15,54]. On the one hand, the CXCR4-*β*-arrestin interaction induces receptor desensitization and endocytosis in the absence of agonist stimulation in HEK cells [55]. On the other hand, *β*-arrestin intensifies CXCL12-induced chemotaxis and enhances the CXCL12-mediated activation of p38 MAPK pathway in HEK cells [54].

Diverse posttranslational modifications of CXCL12 impact on the activation of downstream signaling pathways. For example, Tyr7 nitration affects several CXCL12-induced processes such as ERK1/2 phosphorylation, IP3 production and intracellular calcium release. This modification also reduces monocyte and lymphocyte chemotaxis and pro-inflammatory and tumor-supporting activities of CXCL12 [56]. Furthermore, CXCL12 is post-translationally modified by CD26/dipeptidyl peptidase 4 (DPP4), which cleaves two N-terminal amino acids. The truncated CXCL12 fails to induce intracellular calcium release and chemotaxis of mononuclear and endothelial cells. Moreover, cleavage of CXCL12 by CD26 blocks signaling via ERK1/2 and shifts signaling towards *β*-arrestin recruitment via its scavenger receptor ACKR3 [57].

## 6. The CXCL12/ACKR3 Signaling in Breast Cancer

ACKR3 was first described as a high affinity CXCL12 receptor in T cells [58]. In breast cancer, ACKR3 has been shown to support scavenging of CXCL12. Breast cancer cells expressing ACKR3 internalize and degrade CXCL12 leading to its removal from the environment and to decreased CXCR4 signaling. ACKR3-induced CXCL12 uptake and receptor trafficking depends on *β*-arrestin2 [59]. In addition, endothelial ACKR3 exerts anti-tumor actions in cancer. Healthy endothelial cells show low expression of ACKR3, but it increases in the tumor vasculature in different malignancies including the breast cancer. ACKR3 knockdown in endothelial cells increases the number of circulating tumor cells, recurrence of disease and metastasis suggesting protective role of endothelial ACKR3 in breast cancer against metastasis [60].

Regrettably, ACKR3 can also promote tumor cells survival and high levels of ACKR3 are present in samples from patients with breast cancer [61,62]. Activation of ACKR3 induces VEGF expression and tumor growth but impairs invasion contrary to CXCR4 [63]. Moreover, detrimental actions of ACKR3/CXCR4 dimers have been described in breast cancer. Co-expression of CXCR4 and ACKR3 impairs G*α_i_* mediated signaling and potentiates the *β*-arrestin-dependent MAPK and SAPK signaling. Moreover, co-expression boosts the CXCL12-induced cell migration in metastatic breast cancer cells MDA-MDB-231 [64].

## 7. Inflammation Cuts Both Ways in Cancer

Inflammation plays a crucial role in tumorigenesis and the link between inflammation and cancer was proposed already in the 19th century [65,66]. Cancer-associated inflammation promotes genomic instability, epigenetic modifications, angiogenesis, cancer cell proliferation and invasion [67]. The inflammatory response evoked by immune cells during tumorigenesis can play dual roles. On the one hand, the anti-tumorigenic role of immune cells promotes immunosurveillance and immunological heterogeneity of tumors [66]. Cytotoxic macrophages, neutrophils, mature dendritic cells (DCs) and natural killer (NK) cells help to eliminate both primary and metastatic tumors [67]. On the other hand, tumor-supporting inflammation inhibits anti-tumor immunity and creates the tumor-permissive niche [66,68].

During tumor development, inflammatory cells in co-operation with cancer cells transform tissue architecture in a coordinated manner into the inflammatory tumor microenvironment. Inflammation impacts both the composition of the tumor microenvironment and plasticity of cancer and stromal cells [66]. Early in the carcinogenesis process, immune microenvironment induces tumor-suppressing actions. However, when the tumor becomes invasive it hijacks its environment and turns it into the cancer-promoting niche [2]. Cancer cells have adopted for their own use several molecules of the immune system such as inflammatory chemokines, chemokine receptors and selectins. These signaling molecules attract several subsets of immune cells including neutrophils, immature DCs, macrophages and innate lymphoid cells (ILCs) to fuel inflammation and create supportive microenvironment for cancer growth [65,66,67,69].

## 8. CXCL12 Affects Both Innate and Adaptive Immunity in Cancer

CXCL12 is released by diverse cells in the tumor microenvironment and affects both adaptive and innate immunity in distinct human malignancies [65]. CXCL12 attracts both innate and adaptive immune cells into the tumor boosting its growth [70]. In this section, we describe the effects of CXCL12 on innate and adaptive immune cells in human cancers.

### 8.1. The CXCL12 Effects on Innate Immunity

**Figure 1:**
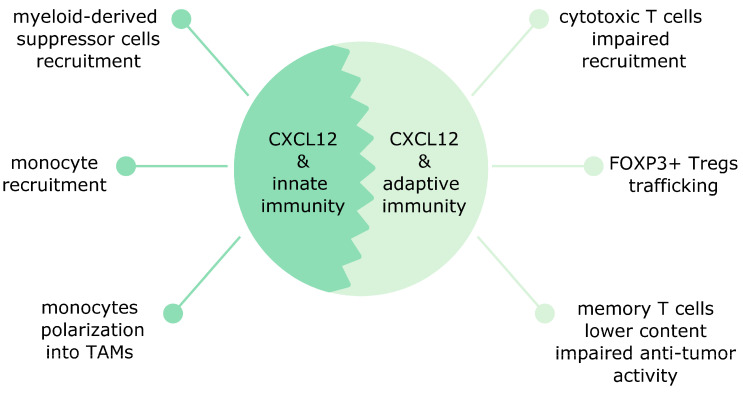
CXCL12 affects both innate and adaptive immunity in cancer. On the one hand, the effects on innate immunity include the recruitment of myeloid-derived suppressor cells, monocyte recruitment and their polarization into tumor-associated macrophages (TAMs). On the other hand, the effects on adaptive immunity are mostly T cells related. CXCL12 attenuates cytotoxic and memory T cells recruitment and increases the content of immunosuppressive FOXP3+ T regulatory cells (Tregs).

In breast cancer, CXCL12 affects innate immunity via promoting monocytes recruitment into tumors (Figure 1). Newly recruited monocytes via CCR2 and CCL2 (also known as monocyte chemoattractant protein-1 (MCP-1)) signaling become motile tumor associated macrophages (TAMs) and later develop into sessile perivascular macrophages. This transformation is unidirectional and is controlled by the CXCL12/CXCR4 axis. Cancer cells-derived TGF-*β* upregulates CXCR4 in motile TAMs and guides them towards CXCL12 released by perivascular fibroblasts. Subsequently, these TAMs associate with blood vessels and induce endothelial barrier leakage. TAMs bring also motile cancer cells and guide them during their journey towards blood vessels [71]. Monocyte recruitment in breast cancer can be controlled by the microRNA pair mir-126/mir-126*. mir-126/mir-126* inhibits CXCL12 expression in stromal cells. This prevents CXCL12-mediated CCL2 release from breast cancer cells and inhibits monocyte and mesenchymal stem cells recruitment and lung metastasis [72]. The CXCR4/CXCL12 pathway in M2 perivascular TAMs has been implicated in breast cancer relapse after chemotherapy in preclinical models. Moreover, a similar M2 subset was present in human breast carcinomas and bone marrow metastases after chemotherapy [73].

In a mouse model of inflammation-driven colorectal cancer, CXCR4 overexpression promotes trafficking of macrophages and myeloid-derived suppressor cells into the colon boosting tumor progression. In addition, CXCL12 attracts immunosuppressive innate immune cells, mostly Ly6Clow monocytes and Ly6G+ neutrophils which predominantly express CXCR4 in the tumor microenvironment. CXCR4 inhibition prevents this infiltration and improves the sensitivity to antiangiogenic therapy [74].

In other types of cancer, the CXCL12/CXCR4 signaling plays an important role in shaping the tumor microenvironment by macrophages. For example, multiple myeloma cells-derived CXCL12 attracts peripheral blood monocytes and transforms them into M2 anti-inflammatory and proangiogenic macrophages [75,76]. These macrophages support growth of multiple myeloma cells and promote resistance to chemotherapy-induced apoptosis. Moreover, they inhibit T-cell proliferation and IFN-*γ* secretion [75]. Likewise, cancer-associated fibroblasts (CAFs)-derived CXCL12 attracts monocytes with the M2 like phenotype and induces their transformation into M2 macrophages in oral squamous cell carcinoma [77]. The CXCL12/CXCR4 signaling also recruits peripheral macrophages into the brain. These macrophages lose their phagocytic activity and fail to phagocytose preneoplastic cells. Similar failure in phagocytosis was observed in microglia suggesting that the activation of CXCL12/CXCR4 can induce tumor-supporting functions in macrophages and microglia already at an early stage of tumor development [78].

### 8.2. The CXCL12 Effects on Adaptive Immunity

In breast cancer, the CXCL12-mediated effects on adaptive immunity are mainly detrimental and affect mostly T cells (Figure 1). High CXCL12 expression in patients with basal-like breast cancer was associated with poor prognosis and high accumulation of Tregs in tumors. This accumulation was mediated by hypoxia-induced CXCR4 [79]. CAFs also contribute to the recruitment of Tregs. In TNBC, the CAF-S1 subset induces via CXCL12 secretion high content of immunosuppressive FOXP3+ Tregs and downregulation of CD8+ T cells content [35]. Although CXCL12 signaling seems to exert rather harmful effects on adaptive immunity in breast cancer, one study reported the opposite observations: CXCL12 overexpression in mouse breast cancer cells was found to induce the CD8+ T cells response and to boost the T cell-mediated cytotoxicity [80].

In ovarian cancer, CXCR4 and CXCL12 overexpression is linked with tumor cell proliferation and the CXCL12/CXCR4 signaling affects the anti-tumor immunity [81]. In mesenchymal high-grade serous ovarian cancer, the CAF-S1-derived CXCL12*β* isoform induced immunosuppression via attraction of CD25+FOXP3+Tregs [82].

An CXCR4 antagonist AMD3100 reduced the intratumoral Tregs content and recruited T-helper cells in a mouse model of ovarian cancer. Moreover, AMD3100 increased T-cell mediated anti-tumor actions in mice [81]. Another study found that CXCR4 inhibition in combination with PD-1 inhibitors upregulated the intratumoral memory T cells content [83]. Thus, CXCR4 blockade in ovarian cancer prevents immunosuppression and improves the outcome of immunotherapy [83].

In other types of malignancies such as osteosarcoma, melanoma and pancreatic cancer, CXCL12 affects both cytotoxic and memory T cells. In osteosarcomas, CXCL12 plays an important role in the homing of cytotoxic T-cells. Since healthy bones express CXCL12, osteosarcomas epigenetically decrease the expression of CXCL12 and impair T cell homing [84]. In contrast, in a mouse model of pancreatic cancer, CAFs-derived CXCL12 prevented tumor infiltration by cytotoxic T cells and induced immune evasion keeping the pancreatic tumors “immunologically cold” [85]. High CXCL12 concentrations drive away T cells from tumors via CXCR4 activation. This phenomenon is called chemorepulsion. In a mouse model of melanoma, tumors expressing high levels of CXCL12 inhibit cytotoxic T cells recruitment. This effect can be reversed by a specific CXCR4 inhibitor. In addition, memory CD8+ T cells fail to elicit the anti-tumor activity in the presence of high levels of CXCL12 [86].

## 9. The CXCL12/CXCR4 Signaling Promotes Fibroblasts Transformation into CAFs and Fuels Breast Cancer Invasiveness

CAFs—the most abundant cell type in the tumor microenvironment—promote angiogenesis, inflammation, chemoresistance and metastasis in contrast to normal fibroblasts, which block tumor formation [2,35]. Moreover, when isolated from human tumors they long remain active. Distinct signaling proteins such as heat shock factor 1 (HSF1), TGF-*β* and CXCL12 have been implicated in transformation of stromal cells in breast cancer.

HSF1 controls transcriptional reprogramming of stromal cells within tumors, it is often activated in CAFs and promotes malignancy. Its knockdown in fibroblasts reduces xenograft tumor growth. HSF1 strong activation is associated with increased tumor grade and poor outcome in breast cancer. HSF1 signaling in fibroblasts activates a specific tumor-supporting transcriptional program via upregulation of TGF-*β* and CXCL12, which act both in autocrine and paracrine manner [87,88]. TGF-*β*-Smad signaling in CAFs induces and maintains their myofibroblast phenotype and induces synthesis of TGF-*β* [87]. Moreover, TGF-*β*1 stimulates the secretion of CXCL12 by CAFs and promotes growth of both TNBC and non-TNBC cell lines [89].

Molecular communication via pro-inflammatory cytokines between breast cancer cells and fibroblasts plays a crucial role in tumor growth and metastasis [90]. For example, IL-7 producing CAFs show a specific oncogenic signature, produce high levels of CXCL12 and physically interact with tumor cells. This leads to the activation of the CXCL12/CXCR4 pathway in breast cancer cells supporting tumor cells stemness and growth in a paracrine manner [91,92]. Moreover, CAFs derived from human breast cancer brain metastasis show higher expression of CXCL12 and CXCL16 in comparison with healthy fibroblasts and CAFs from primary breast tumors. They are stronger inducers of cancer cell migration. High levels of CXCL12 and CXCL16 in CAFs from human brain cancer metastasis attract breast cancer cells via the CXCR4-CXCL12 and CXCR6-CXCL16 pathways [33].

CAFs together with endothelial cells generate local CXCL12 gradients to attract CXCR4+ progenitor cells such as endothelial progenitor cells, enhancing tumor angiogenesis [92]. Furthermore, CAFs-derived CXCL12 decreases endothelial barrier integrity, enhances vascular permeability and growth of leaky tumor vasculature promoting distant breast cancer metastasis [32]. Other studies found similar results on the effect of CXCL12 on endothelium in breast cancer [93,94]. However, recent studies exist that suggest the protective role of CXCL12 for the endothelial integrity [95,96]. This discrepancy could be explained by different types of endothelial cells used in the different studies and also the different experimental settings. Moreover, different CXCL12 isoforms can exert differential effects on the endothelial integrity [94].

CAFs-derived CXCL12 plays specific roles in TNBC. It enhances invasiveness of TNBC cells via ERK1/2 activation [97]. Furthermore, CXCL12 boosts TNBC cells migration via cytoskeletal rearrangements [98]. These effects can be blocked by a specific CXCR4 inhibitor, suggesting that disrupting the interactions with tumor microenvironment might be a promising therapeutic strategy for TNBC that lacks a targeted treatment [97].

## 10. The CXCL12/CXCR4 Pathway Induces Breast Cancer Motility

Cell motility and migration belong to indispensable cell behaviors and contribute to cancer metastasis, which remains the biggest challenge in treatment of the disease [99].

Many studies show that small G proteins such as Rho, Rac and Cdc42, which regulate the cytoskeleton rearrangement, play an important role in the CXCL12-induced migration. Cdc42 and Rac1 regulate formation of filopodia and lamellipodia formation, whereas RhoA controls stress fibers and focal adhesion [100]. In MCF-7 cells, the Rac1 specific inhibitor NSC23766 blocks the CXCL12-induced chemotaxis. This inhibitor shows dual secondary effects on the CXCR4 signaling. It acts as an activator in cAMP assays whereas it is inhibitory in migration and calcium release assays [101]. Another study showed that heregulin, a ligand for Erb3 and Erb4 receptors, sensitizes breast cancer cells for CXCL12 mediated Rac1 activation. Pre-treatment with heregulin induces stronger migration in response to CXCL12 and higher HIF-1*α*-induced CXCR4 expression [102]. In MDA-MB-231 cells, CXCL12/CXCR4 signaling induces actin polymerization and invasion [25]. However, another study found that the effect of CXCL12 on migration is concentration dependent in TNBC cells. The underlying mechanism relies upon the activation of specific small G proteins. Low concentrations of CXCL12 induce overexpression and activation of RhoA associated with actin polymerization and cell locomotion. In contrast, higher concentrations of CXCL12 promote Rac1-controlled cell adhesion [103].

The epithelial–mesenchymal transition (EMT) allows cancer cells to gain migratory and metastatic properties. Recent studies indicate important role of CXCL12 in EMT induction. DPP-4, which cleaves CXCL12, prevents CXCL12induced EMT in breast cancer. DPP-4 inhibition activates the CXCL12/CXCR4 and mTOR signaling. This induces EMT and migration in both TNBC and nonTNBC breast cancer cells and therefore promotes metastasis. Moreover, DPP4 knockdown promotes metastasis in vivo [104]. The heavy subunit of ferritin (FHC) has also been implicated in CXCL12-induced EMT and proliferation in breast cancer cells. FHC silencing induces mesenchymal phenotype and enhances migration and proliferation via CXCL12/CXCR4 activation and ROS induction. This leads to E-cadherin downregulation and induction of vimentin, indicating the EMT phenotype [105]. Another study showed that CXCL12-induced EMT can be inhibited by a GPR54 ligand kisseptin-10 [106].

## 11. The Role of CXCL12 and CXCR4 in Breast Cancer Metastasis

**Figure 2:**
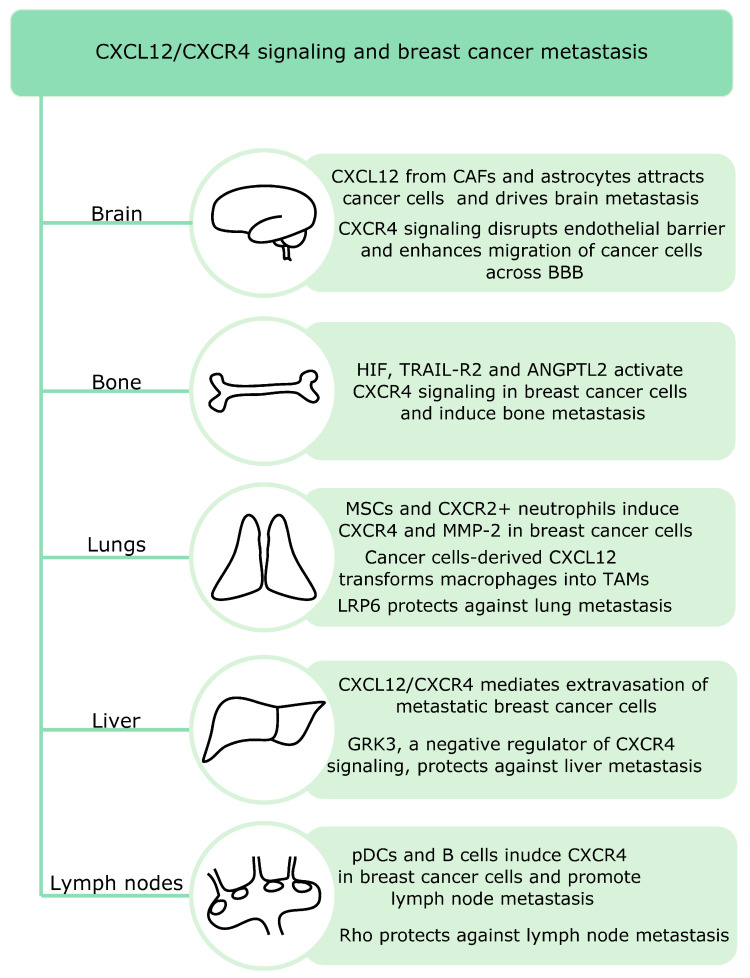
The CXCL12/CXCR4 signaling controls all types of breast cancer metastasis. In the brain, CXCL12 released by cancer-associated fibroblasts (CAFs) and astrocytes attracts cancer cells and facilitates their migration across the blood–brain barrier (BBB). In the bones, diverse signaling proteins such as hypoxia-inducible factor (HIF), TNF-related apoptosis-inducing ligand receptor 2 (TRAIL-R2) and angiopoietin-like protein 2 (ANGPTL2) can activate CXCR4 in breast cancer cells. In the lungs, crosstalk between mesenchymal stromal cells (MSC2) and neutrophils induces metastasis-related genes such as CXCR4 and matrix metalloproteinase-2 (MMP-2). Moreover, cancer cells-derived CXCL12 induces transformation of macrophages into tumor-associated macrophages (TAMs). The low-density lipoprotein receptor-related protein 6 (LRP6), a crucial component of the Wnt canonical pathway, protects against lung metastasis. In the liver, CXCL12/CXCR4 is involved in extravasation of cancer cells. The G-protein-coupled receptor kinase 3 (GRK3) plays a protective role against liver metastasis. Both innate and adaptive immune cells have been implicated in lymph node metastasis. Rho, a member of small G proteins family, can protect against this type of breast cancer metastasis.

CXCR4 remains crucial in breast cancer metastasis pattern to the lungs, liver, bones and lymph nodes (Figure 2) since these metastatic niches express high levels of its ligand CXCL12 [107].

Recent studies suggest that both innate and adaptive immune cells are involved in lymph node metastasis in breast cancer. Breast cancer lymph node positive patients show increased infiltration of plasmacytoid DCs and this correlates with the percentage of CXCR4 positive cells. pDCs secrete TNF, which induces CXCR4 expression in breast cancer cells. Moreover, high expression of CXCL12 is present in those patients [34]. Another study showed that primary tumors induce accumulation of B cells in the tumor-draining lymph nodes and release of pathogenic IgG that induces CXCR4 expression in cancer cells. High serum IgG levels also correlate with lymph node metastasis in patients with breast cancer [108]. Signaling by small signaling G proteins such as Rho can protect against lymph node metastasis. RhoA inhibition in primary tumors enhances lymph node invasion. Moreover, RhoA blockade affects tumor microenvironment and increases macrophage and fibroblast infiltration via activation of the chemokine CCL5-CCR5 and CXCL12CXCR4 signaling [109].

Breast cancer metastatic relapse can occur many years after treatment and bone marrow is a major site of disease relapse. Price et al. studied the mechanisms underlying bone marrow microvasculature entry by the disseminated breast cancer cells and found that dormant and proliferating cells occupy distinct niches in the bone marrow. Dormant cells remain in the E-selectin and CXCL12-rich perisinusoidal vascular regions. E-selectin allows the entry of breast cancer cells into the bone marrow, whereas the CXCL12/CXCR4 signaling anchors breast cancer cells in the microenvironment [110]. Another study showed that activation of HIF signaling in osteoprogenitor cells increases bone mass and promotes breast cancer metastasis to the bones. Moreover, HIF signaling in osteoblast-lineage cells promotes breast cancer cells growth and dissemination in remote tissues such as lungs via increase in blood levels of CXCL12. Thus, bones might regulate systemic tumor microenvironment and growth [111]. In addition, tumor cell-derived angiopoietin-like protein 2 (Angptl2) induces CXCR4 and activates CXCL12/CXCR4 signaling in breast cancer cells, leading to bone metastasis. In mouse xenografts models, Angplt2 inhibition reduces bone metastasis. Moreover, the expression of Angptl2 and CXCR4 positively correlated in patient samples [112]. TRAIL-R2, which belongs to the TNF receptor superfamily, has also been implicated in bone metastasis in breast cancer. TRAIL-R2 knockdown in MDA-MB-231 cells downregulates CXCR4 expression and migration and blocks the capability to metastasize to the bones. Moreover, it increases E-cadherin expression [113].

Lung metastasis remains a significant cause of deaths in patients with breast cancer and the CXCR4/CXCL12 pathway is a key player in this process. Both stromal and immune cells can induce lung metastasis in breast cancer. Activated mesenchymal stromal cells play an important role in murine models of breast cancer lung metastasis. Upon challenge with TNF, these cells express CCL5, CCR2 and CXCR2 ligands. The CXCR2 ligands (CXCL1, -2 and -5) recruit CXCR2+ neutrophils into the tumor microenvironment, which interact with cancer cells and induce expression of metastasis-involved genes such as CXCR4 and MMP-2 [114]. Moreover, POU1F1-induced CXCL12 in breast cancer cells promotes recruitment and polarization of macrophages into TAMs. The latter act to enhance tumor growth, angiogenesis and metastasis to the lungs [115]. Another study found that LRP6, a key component of canonical Wnt signaling, can suppress lung metastasis both in vitro and in vivo. LRP6 ectodomain blocks CXCR4/CXCL12 induced lung metastasis via binding to CXCR4. The onset of metastasis decreases LRP6 levels in both murine and human serum from individuals with breast cancer. Moreover, low LRP6 expression worsened already poor prognosis of patients with breast cancer and high CXCR4 levels [116].

Brain remains a more frequent target organ for TNBC than for luminal breast cancers [2]. Astrocytes, which regulate both innate and adaptive immune responses upon injury in the central nervous system, have been involved in brain metastasis. They promote breast cancer brain metastasis via CXCL12 secretion. CXCL12 downregulates the expression of Kiss1 in breast cancer cells, a gene associated with metastasis inhibition. The expression of Kiss1 in human breast cancer specimens inversely correlates with levels of matrix metalloproteinase-9 (MMP-9) and IL-8—genes implicated in metastatic invasion [117]. CAFs have also been implicated in brain metastasis. CAFs from brain metastasis are stronger inducers of cancer cell migration. High levels of CXCL12 and CXCL16 in CAFs from human brain cancer metastasis attract breast cancer cells via the CXCR4/CXCL12 and CXCR6/CXCL16 pathways [33]. Furthermore, the CXCL12 signaling in endothelial cells increases their permeability and enhances migration of breast cancer cells across the blood brain barrier [118].

Liver metastasis is more common for TNBC and HER2+ than luminal breast cancers [2]. The CXCL12/CXCR4 signaling is involved in extraversion of metastatic breast cancer cells in the liver [119]. Billard et al. found that G protein-coupled receptor kinase 3 (GRK3)—a negative regulator of CXCR4 signaling—protects against liver metastasis. Decreased GRK3 expression correlates with basal-type breast cancer and liver metastasis in patients and alterations in GRK3 expression influence both migration and invasion of TNBC cells [120].

In summary, CXCL12/CXCR4 signaling acts as a master regulator of breast cancer metastasis and mediates metastasis to all target organs such as brain, lungs, lymph nodes, liver and bones. This signaling affects many cell types including breast cancer cells, endothelial cells, stromal cells and astrocytes to induce metastasis in a coordinated manner. Favorably, specific types of signaling proteins such as Rho, LRP6 and GRK3 have been described as protective against lymph node lung and liver metastasis.

## 12. CXCL12/CXCR4-Targeted Therapies against Breast Cancer

Since the CXCL12/CXCR4 cascade plays important roles in many aspects of breast cancer tumorigenesis, treatments aiming to inhibit this pathway might provide valuable therapeutic tools. Multiple strategies aiming at blocking CXCR4 expression in breast cancer have been proposed. For example, peroxisome proliferator-activated receptor (PPAR)-*γ*—a member of the nuclear receptor superfamily—can inhibit CXCR4. Upon ligand activation PPAR-*γ* recruits transcriptional corepressor SMRT onto the CXCR4 promoter in both stromal and breast cancer cells. In addition, a PPAR-*γ* agonist—rosiglitazone—reduces migration of both CAFs and breast cancer cells [121]. In addition, a novel PPAR-*γ* agonist VSP-17 inhibits TNBC metastasis via induction of E-cadherin [122]. Thymoquinone (TQ) is another compound that blocks CXCR4 expression in TNBC cells. TQ reduces NF-*κ*B binding to the CXCR4 promoter. This result correlated with decreased migration and invasion of MDA-MB-231 cells in response to CXCL12 and lower lung, brain and bones metastases in vivo [123]. In addition, saikosaponin A, anti-VEGF Ab and gap junction inhibitor, reduces the expression of CXCR4 in breast cancer cells [124,125].

Other CXCR4-targeting therapeutic strategies in breast cancer aim at inhibiting CXCR4-dependent migration, invasion and metastasis. CXCR4 antagonist POL5551 reduces migration of TNBC breast cancer cells in vitro. In combination with a microtubule inhibitor eribulin, POL5551 reduces metastasis and prolongs survival in vivo [126]. In another study, lidocaine blocked the CXCR4 signaling and decreased intracellular calcium release, cytoskeleton remodeling and cell migration in MDA-MB-231 cells [127]. The CXCR4-targeted dendrimers encapsulating doxorubicin also reduce CXCL12-induced migration of BT-549 and T47D breast cancer cells [128]. Similarly, low molecular weight heparin (LMWH)-taurocholate conjugated with tetrameric deoxycholic acid, namely LHTD4, inhibits TGF-*β*1 and CXCL12 mediated migration and invasion of breast cancer cells [129]. Saikosaponin A reduces migration, colony formation and proliferation of breast cancer cells. In vivo, saikosaponin A decreases lung metastasis and expression of metastasis-related genes such as MMP-2 and MMP-9 [124].

Dimerization seems to be another effective strategy to block the CXCL12/CXCR4 dependent migration in breast cancer. For example, the CXCL12-CXCL4 heterodimers prevent migration of TNBC cells. A CXCL4-derived peptide homologous to the binding interface, identified by NMR, mimicked the activity of the native CXCL4. This suggests that peptides imitating CXCL12-CXCL4 interactions might be a novel therapeutic strategy to prevent CXCL12-induced breast cancer cell migration [130]. CXCR4 also forms both homo- and heterodimers with other GPCRs, leading to enhancement or decrease in their activity depending on the interacting partner. CXCR4 can interact with cannabinoid receptor 2 in TNBC cells. This interaction reduces CXCR4-mediated ERK1/2 phosphorylation and chemotaxis suggesting that the cannabinoid signaling might decrease CXCR4 signaling and possibly breast cancer progression [131].

Although CXCL12/CXCR4 inhibition brings promising results in breast cancer trials, systemic effects of blocking CXCR4 such as alterations in the spleen remain poorly understood [132,133]. CXCR4 antagonists including AMD3100 could also exhibit potential against bone marrow metastasis in breast cancer. However, the CXCR4 inhibition affects osteoclastogenesis and enhances tumor growth in the bones [134]. In addition, CXCR4 effects in breast cancer depend on the disease subtype. In patient-derived xenografts, CXCR4 inhibitors AMD3100 and TN14003 block tumor growth and metastasis in both herceptin-sensitive and herceptin-resistant HER2+ breast cancer. In contrast, these inhibitors failed to show beneficial effects in TNBC [135].

Overall, several strategies to block CXCL12/CXCR4 signaling in breast cancer have been proposed including PPAR-*γ* agonists, dimerization with other receptors or chemokines and specific CXCR4 inhibitors including AMD3100. Since AMD3100 shows clinical efficacy in hematological cancers such as leukemia, this result provoked clinical applications in other cancers. However, the discussion about the use of CXCR4 inhibitors is still ongoing in the field. Specifically, biological effects caused by long-term use remain insufficiently understood [136].

## 13. The CXCL12/CXCR4-induced Therapy Resistance in Breast Cancer

Diverse mechanisms underlying the CXCL12/CXCR4-induced therapy resistance have been reported in breast cancer. For example, the CXCL12/CXCR4 signaling boosts the activity of PI3K and ERK promoting tumor growth and drug resistance in TNBC cells [137]. Moreover, CXCR4 overexpression activates ERK1/2 and AKT via platelet-derived growth factors A and B [138]. Another study found that ERK activation via a member of small G proteins Rac1 increases nucleotide metabolism in breast cancer cells and protects against chemotherapy-induced cytotoxicity and DNA damage. Rac1 is also upregulated in chemoresistant breast cancer and correlates with poor prognosis [139].

Cytoskeletal proteins such as cytokeratin-19 have been implicated in development of CXCL12/CXCR4-dependent drug resistance. Inhibition of cytokeratin-19 in TNBC cells prompts proliferation, migration and drug resistance via CXCR4 upregulation. Moreover, low cytokeratin-19 expression in patient samples correlates with poor prognosis [140]. Another study evaluated the link between cytokeratin-19 expression in cancer cells during the follow-up period after treatment and reached a different conclusion. The presence of cytokeratin-19 positive circulating tumor cells in the first five years of follow-up indicates the presence of therapy resistant residual disease and correlates with increased risk of late relapse [141].

The CXCL12/CXCR4 signaling in breast cancer cells also induces resistance to immune checkpoint blockers. Immune checkpoint blockade combined with nabpaclitaxel increases progression-free survival in patients with TNBC. However, fibrotic tumor microenvironment can suppress the anti-cancer response of immune cells and therefore a large subset of patients fail to benefit from this treatment. CXCR4 inhibition with AMD3100 decreases desmoplasmia, fibrosis, decompresses blood vessels, increases cytotoxic T cells infiltration and decreases immunosuppression in murine models of breast cancer. Thus, CXCL12/CXCR4 blockade boosts the efficacy of immune checkpoint blockers in preclinical models of breast cancer [142].

In summary, CXCR4 overexpression in breast cancer induces ERK activation, proliferation and drug resistance. Furthermore, CXCL12/CXCR4 axis supports fibrotic tumor microenvironment and prevents therapeutic effects of immune checkpoint blockers.

## 14. Conclusion and Future Outlook

**Figure 3:**
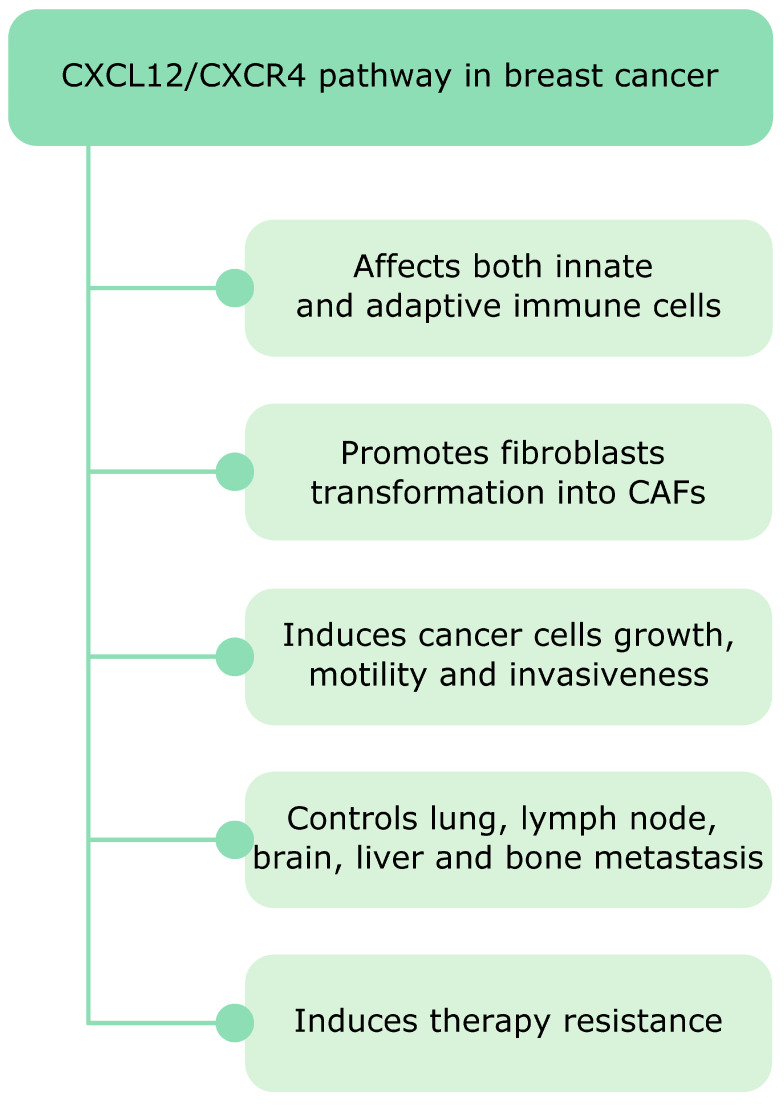
Various aspects of how the CXCL12/CXCR4 signaling controls breast cancer tumorigenesis. It affects both innate and adaptive immunity and induces transformation of fibroblasts into CAFs. Moreover, CXCL12/CXCR4 is implicated in disease progression and metastasis to distant organs such as lungs, lymph nodes, brain, liver and bones. Finally, activation of this signaling pathway might lead to therapy resistance.

After the discovery of the CXCR4 involvement in leukemia its role in cancer has become a widely studied research topic. High levels of CXCR4 have been found in diverse human cancers and play mostly detrimental roles facilitating disease progression and decreasing overall survival [24]. A plethora of studies show that CXCR4, the chemokine receptor most commonly overexpressed in cancer, together with its natural chemokine ligand CXCL12 acts as a master regulator of breast cancer tumorigenesis (Figure 3). The CXCL12/CXCR4 signaling affects both innate and adaptive immunity in breast cancer. For example, it induces transformation of monocytes into tumor-supporting TAMs and promotes immunosuppression via Tregs recruitment. Moreover, CXCL12 affects stromal cells and controls transformation of healthy fibroblasts in CAFs, which also release CXCL12 and further fuel tumor growth. Finally, the CXCL12/CXCR4 signaling induces breast cancer cell motility and is involved in all types of breast cancer metastasis.

The CXCL12/CXCR4 signaling has also been a therapeutic target in breast cancer research. Recent studies propose several strategies to inhibit this signaling including specific CXCR4 inhibitors, which block its downstream effects such as migration and metastasis, with AMD3100 discovered as contamination during commercial preparation of anti-HIV compounds [143]. This highly specific CXCR4 inhibitor was approved more than a decade ago for treatment of multiple myeloma and non-Hodgkin’s lymphomas [144]. Current research suggests it might also be beneficial in other malignancies including breast cancer to fight resistance to immunotherapies and radiation therapies [1,19,145].

As future perspectives, we suggest two directions. First, we believe that computational approaches aiming to explore high-throughput data will generate novel basic knowledge about CXCL12/CXCR4 signaling in breast cancer. For example, open-access databases such as TCGA can help to uncover abnormalities and novel druggable targets. Moreover, other computational approaches such as information theory that has recently been applied in the field of biological signal transduction could be beneficial to study the role of the CXCL12/CXCR4 pathway in breast cancer pathogenesis [146]. Second, we suggest using diverse high-throughput technologies to evaluate the current status of CXCL12 signaling in patients with breast cancer during treatment. Considering that therapies can alter the CXCL12/CXC4 status and therapy sensitivity [74], such an evaluation would enable modification of treatment to ensure the best outcome with less toxicity. This approach seems specifically appealing in the context of challenges of personalized medicine. Although these are formidable tasks, we believe they might provide benefits for patients suffering from this devastating disease.
